# Effect of Helicobacter Pylori Infection on Nutritional Status in Polish Teenagers

**DOI:** 10.1155/2021/6678687

**Published:** 2021-03-30

**Authors:** Anna Szaflarska-Popławska, Anetta Soroczyńska-Wrzyszcz

**Affiliations:** ^1^Department of Pediatric Endoscopy and Gastrointestinal Function Testing, Collegium Medicum in Bydgoszcz, Nicolaus Copernicus University in Toruń, Poland; ^2^Department of Pediatrics, Gastroenterology, Neurology, Endocrinology and Diabetology, Dr Władysław Biegański Regional Specialist Hospital in Grudziądz, Poland

## Abstract

**Purpose:**

Data on an association between *Helicobacter pylori* (*H*. *pylori*) and nutritional status in children are conflicting. We designed a large-sampled prospective community-based study to examine the differences in average body indices among Polish teenagers depending on their *H*. *pylori* status.

**Methods:**

From September 2008 to June 2015, 3067 second junior high school students aged between 13 and 17 years (mean age: 14.5) from 11 randomly selected schools from Grudziadz, Poland, were recruited. For the cohort, ^13^C urea breath test for current *H*. *pylori* infection was performed and data on anthropometric measurements and sociodemographic characteristics were collected. *Z* scores of height for age (HAZ), weight for age (WAZ), and BMI for age (BMIZ) were calculated.

**Results:**

The *H*. *pylori* colonisation rate was 23.6% with no gender difference. Compared to noninfected, *H*. *pylori* infected had significantly lower mean WAZ (0.0085) and BMIZ scores (*p* = 0.0246). Univariate linear regression models showed that living in the old town district and consumption of tap water were negative predictors of HAZ, living in the old town district, using collective catering facilities, and *H*. *pylori* infection were negative predictors of WAZ, and using collective catering facilities and *H*. *pylori* infection were negative predictors of BMIZ. In the multiple regression analyses, living in the old town district (*p* = 0.0039), using collective catering facilities (*p* < 0.0001), and *H. pylori* infection (*p* = 0.0269) were confirmed to be independently associated with lower WAZ, whereas using collective catering facilities (*p* < 0.0001) and *H. pylori* infection (*p* = 0.0265) were confirmed to be independently associated also with lower BMIZ.

**Conclusion:**

Our finding confirms the evidence on independent negative influence of *H*. *pylori* infection on nutritional status in Polish teenagers.

## 1. Introduction

*Helicobacter pylori* (*H. pylori*) is the most common chronic bacterial infection as it affects approximately one-third of children worldwide. Most infections are commonly acquired in childhood. Furthermore, in most, if not all, infected individuals, *H. pylori* causes chronic gastritis that in children seems to be mainly antrum-predominant. Unlike adults, most infected children remain asymptomatic. Only a minority develop duodenal and gastric ulcer [1]. The question whether *H. pylori* infection plays a role in nutritional status of affected children in numerous research studies has been examined, but results were inconsistent. Given these controversial data, we designed a large-sampled prospective community-based study to examine the differences in average body indices among Polish teenagers depending on their *H. pylori* status.

## 2. Materials and Methods

The study was conducted in Grudziadz, an industrial city in north-central part of Poland with a population about 100,000 residents. A selection of the city for the research was associated with the source of financial coverage (the City Council of Grudziadz) and the city-level socioeconomic variation. The city is divided into 7 new districts and 6 old districts. Living in the old district reflects a combination of lower household income, higher unemployment rate, and lower educational level of parents, as well as poorer housing condition and lower level of hygiene and sanitation when compared to the new city district.

In this prospective cross-sectional study conducted between September 1, 2008, and June 30, 2015, 3375 second junior high school students from eleven randomly selected secondary schools from Grudziadz, Poland, were recruited. A total of 3241 participants aged between 13 and 17 years (mean age: 14.5), who signed an informed consent, were investigated for body height, weight, and *H. pylori* status. Moreover, data on sociodemographic and hygiene practices with use of a self-completed questionnaire were collected. The questionnaire was divided into three main sections as follows: (a) sociodemographic variables (gender, age, residence area, and household crowding), (b) clinical symptoms, and (c) hygiene practices (consumption of raw vegetables, raw meat, tap water, using collective catering facilities, washing hands after coming back home, using a toilet, contact with animals and before meal, owing a dog, a cat, or both). Of these 3241 subjects, 1892 (58.4%) were girls and 1349 (41.6%) were boys. Of these 3241 subjects, ^13^C urea breath test results were obtained from 3067 participants, who were eligible for the final analysis.

In the group, 67 of 3067 (2.2%) were older than 16 years and 1382 (45.1%) lived in old city districts. Collective catering facilities were used by 734 (27.5%) questionnaire responders. Consumption of raw vegetables, raw meat, and tap water was self-reported by 1735 (56.6%), 365 (11.9%), and 835 (27.2%) participants, respectively. Hand washing after coming back home, before meal, after using the toilet, and after contact with animals was not practiced by 842 (27.5%), 590 (19.2%), 170 (5.5%), and 1053 (34.4%) participants, respectively. Owing a cat, a dog, or both was reported by 336 (11%), 1070 (34.9%), and 259 (8.4%) questionnaire responders, respectively.

For the cohort, ^13^C urea breath test (UBT) for current *H. pylori* infection was performed using HeliFAN plus analyzer (Fischer Analysen Instrumente GmbH). The protocol of the test was consistent with the manufacturer's specifications. A validation of the test was conducted once a year with the use of samples of known gas concentration. The accepted variation in the test results between tested and standardized samples was below 10% as recommended. Breath samples before and 30 minutes after the intake of 75 mg of ^13^C-labeled urea were collected. The final result was expressed as the difference between the two scores, delta over baseline (DOB). The cut-off point was 4.0. A result equal to or higher than 4.0 DOB was considered positive for *H. pylori* infection. All participants tested positive for UBT were offered upper gastrointestinal endoscopy with multiple gastric biopsies. All children with histologically confirmed *H. pylori*-related lesions were scheduled for the empiric eradication therapy.

### 2.1. Anthropometric Measurements and Indices

Anthropometric measurements were performed by trained registered nurses. Body weight was measured using a digital scale (calibrated before use), and body height was measured by a stadiometer. The calculation of BMI (body mass index) was performed on the basis of height and weight measurements (kg/m^2^). Height-for-age, weight-for-age, and BMI-for-age were expressed as *Z* scores. *Z* scores of height for age (HAZ), weight for age (WAZ), and BMI for age (BMIZ) were calculated using the formula: *Z* score = (observed value–median value of the reference population)/standard deviation value of reference population. The calculations were based on the 2010 Polish growth reference charts.

### 2.2. Statistical Analysis

The mean and the standard deviation of HAZ, WAZ, and BMIZ scores for *H. pylori*-infected and *H. pylori*-noninfected groups were calculated. Due to great differences in number of participants in the groups, *U*-Mann–Whitney test to compare HAZ, WAZ, and BMIZ scores between groups was used. A linear regression was used with HAZ, WAZ, and BMIZ as the outcome, and the independent variables were age, sex, and selected sociodemographic and hygienic characteristics. The selected independent variables were combined to generate the regression function. Using a stepwise approach through backward elimination, beginning with a model that included all predictors, candidate predictors from the saturated model based on their statistical significance (Wald test *p* > 0.05) were excluded. Nonlinear relations between outcome and continuous predictors were considered by identifying, at each iterative step of the stepwise process, the best fitting fractional polynomial terms. This model development process led to a final model for the prediction of HAZ, WAZ, and BMIZ based on the selected predictors along with their corresponding estimated *β* coefficients and the associated intercept term. Statistical analyses were performed using TIBCO Statistica® 13.3.0 and R, version 3.3.2 (R Foundation for Statistical Computing, Vienna, Austria). All tests were considered statistically significant at *p* value less than 0.05.

The study was approved by Bioethics Committee of Nicolaus Copernicus University in Toruń (KB 490/2008). The informed consent was obtained from each study participant and signed by a parent/caregiver for children below 16 years and by both a parent and a child aged 16 years and older.

## 3. Results

Seven hundred and twenty-three participants (23.6%) had a positive ^13^C UBT results (Hp (+)) and 2344 subjects (76.4%) had a negative ^13^C UBT results (Hp (-)) with no statistically significant difference in ^13^C-UBT positivity between males and females.

The mean (*M*) and standard deviation (SD) of HAZ, WAZ, and BMIZ scores in children tested positive and negative for *H. pylori* are presented in Table 1. Compared to noninfected, *H. pylori* infected had lower mean HAZ, WAZ, and BMIZ scores with WAZ and BMIZ scores being statistically significant.

Univariate linear regression models showed that living in the old town district and consumption of tap water were negative predictors of HAZ, whereas washing hands after coming home, toilet, and contact with animals were positively associated with HAZ. A negative association between HAZ and *H. pylori* positivity was of borderline statistical significance (Table 2). Multiple analyses revealed that only one variable, i.e., washing hands after toilet was independently associated with HAZ (Table 3).

In the univariate linear regression models, living in the old town district, using collective catering facilities and *H. pylori* infection were negative predictors of WAZ (Table 4). These three variables were confirmed in the multiple regression analyses to be independently associated with lower WAZ. Male sex was the only factor positively associated with WAZ in both univariate linear and multiple regression analyses (Table 3).

Univariate linear regression models showed that using collective catering facilities and *H. pylori* infection were negative predictors of BMIZ whereas male sex and consumption of tap water were positively associated with BMIZ (Table 5). Multiple regression analyses revealed that all these four variables were independent predictors of BMIZ (Table 3).

## 4. Discussion

Human growth is complex and depends on factors that may also be associated with *H. pylori* acquisition, such as socioeconomic status and hygiene practices. The association between nutritional status and *H. pylori* infection remains controversial. Some studies showed negative effect of *H. pylori* colonisation on both height and weight being affected [2–5] or only height [6, 7] or only weight [8, 9] being affected. However, other studies suggested no influence of *H. pylori* infection on growth [10] or even a positive correlation between the infection and a high BMI [11]. The inconsistency may arise from differences in sample size, testing methodology of *H. pylori* infection, and, most of all, study population (developed versus developing countries and different age groups).

In this large-sampled prospective community-based study, we showed that WAZ and BMIZ scores were significantly lower in 13-17-year-old Polish teenagers tested positive for *H. pylori* infection than their noninfected counterparts. Furthermore, this negative association between *H. pylori* infection and both WAZ and BMIZ scores was independent of sociodemographic variables and different hygienic practices. These results support a negative relation between weight and BMI and *H. pylori* infection and suggest that except for improvements in socioeconomic and hygienic conditions, in children with growth disturbances, *H. pylori* testing should be offered. This is in agreement with a Peruvian [12] and Colombian [3] studies that found a significant and permanent effect of *H. pylori* infection on weight among children from low socioeconomic background. According to the systematic review by Lender et al. [13], there is a strong inverse association between prevalence of *H. pylori* infection and overweight and obesity in developed countries, which also confirms the negative effect of *H. pylori* on body weight. In contrast with these findings, in the largest pediatric study on the subject to date, differences with regard to both body weight and body mass index between infected and noninfected children were not significant. However, in this study, boys with *H. pylori* infection had significantly lower weight than those noninfected [4].

In this study, children tested positive for *H. pylori* infection had lower HAZ score, but the difference was not statistically significant. This finding may suggest that weight gain is influenced by *H. pylori* infection in an earlier time period, compared to height. It can be hypothesized that with exposure time elongation, the effect of *H. pylori* infection on height would become more prominent. This hypothesis is consistent with a prior cohort study of Kocaoglu et al. [14] who concluded that the negative effect of the infection on both height and weight is evident as the duration of exposure is prolonged. Several previous studies indicated that chronic *H. pylori* infection may negatively influence growth [4–6] and can be associated with short stature in children [7]. In contrast with these findings, some pediatric studies reported no link between height and *H. pylori* infection [15, 16].

Our study and other well-designed studies support the notion that *H. pylori* might negatively affect physical growth, likely after the establishment of persistent infection. It is no clear by which mechanism *H. pylori* may impair physical growth; nonetheless, there are multiple plausible explanations. It has been shown that *H. pylori*-related inflammation can affect the levels of neuroendocrine gastric hormones such as ghrelin and leptin. They play a crucial role in regulation of eating behaviour and energy expenditure. Ghrelin is a peptide hormone well known for its growth hormone-releasing and appetite-stimulating effect. In a systematic review published in 2011, the significantly lower circulating ghrelin concentration in *H. pylori*-positive subjects compared to those not infected has been conclusively confirmed [17]. However, the effect of *H. pylori* infection on plasma ghrelin level has been demonstrated mainly in adults with the fundus-predominant gastritis. In children, in whom *H. pylori*-related gastritis is mainly of antrum-predominant type, ghrelin secretion impairment is not expected [5]. Some studies have also revealed an association between H. pylori infection in children and increased serum concentration of leptin that has anorexigenic actions [18].

Another explanation is that *H. pylori* may induce a decrease in iron stores. Children with iron deficiency may have reduced appetite that may affect their nutritional intake and their overall physical status. In addition, some studies have shown an association between chronic *H. pylori* infection and disturbed absorption of many nutrients and vitamins, which in turn impairs childhood growth [19]. The current meta-analysis documented a significantly increased likelihood of iron deficiency anemia in *H. pylori*-infected individuals compared to uninfected group. The association was stronger in children, for whom a 2-fold higher prevalence of iron deficiency anemia was observed [20]. However, some studies did not confirm the association between *H. pylori* positivity and the dietary intake [21].

This study has both limitations and strengths. The strengths of our study include its prospective design and a very large sample of the population. It is one of the largest studies concerning height and weight in relation to *H. pylori* infection and the largest conducted in Poland. Furthermore, for the diagnosis of *H. pylori* infection, we used the ^13^C urea breath test that remains a most reliable noninvasive test with the highest diagnostic odds ratio when compared to ^14^C urea breath test, serology, and stool antigen [22].

The main limitation of the study is the lack of detailed socioeconomic data. We analyzed the patients' place of residence as a surrogate marker for socioeconomic status. It can be misleading, as living in old city district might not be inseparably associated with poor hygienic practices and other factors playing a role in *H. pylori* acquisition.

Secondly, the study was conducted among teenagers from small region in Poland; therefore, conclusions drawn based on its result may not be universally applicable. However, we recruited a cohort of individuals from fairly heterogeneous socioeconomic background, which enables us to assess the effect of *H. pylori* infection on the nutritional indices in individuals from different socioeconomic conditions.

Finally, the present study provides no insight into the pathogenesis of the inverse correlation between anthropometric indicators of nutritional status and *H. pylori* infection.

## 5. Conclusion

In conclusion, by employing the most accurate noninvasive diagnostic method for *Helicobacter pylori* detection, our finding adds to the evidence on independent negative influence of *H. pylori* infection on nutritional status in Polish teenagers.

## Figures and Tables

**Table 1:** Comparison of HAZ, WAZ, and BMIZ scores in *Helicobacter pylori-*negative and -positive children.

Variable	Hp	*N*	Mean	SD	*p*
HAZ	Hp (-)	2344	0.22	1.0593	0.0602
	Hp (+)	723	0.14	1.1600	
WAZ	Hp (-)	2344	0.09	1.0028	0.0085
	Hp (+)	723	-0.02	1.0121	
BMIZ	Hp (-)	2344	0.01	0.9918	0.0246
	Hp (+)	723	-0.08	0.9451	

**Table 2:** Unadjusted parameter estimate (*β*) with standard errors (SE) of HAZ according to sociodemographic and hygienic characteristics.

Variable	*β*	SE	*p*
Male sex	-0.03	0.02	0.1049
Age			
13 years	0.03	0.01	0.1199
14 years	-0.11	0.19	0.5697
15 years	-0.17	0.18	0.3679
16 years	-0.11	0.06	0.0614
17 years	-0.01	0	0.9378
Living in the old town district	-0.06	0.02	0.0006
Consumption of raw vegetables	0.01	0.02	0.3932
Consumption of raw meat	-0.03	0.02	0.1293
Consumption of unboiled water	-0.04	0.02	0.0183
Using collective catering facilities	-0.02	0.02	0.3689
Washing hands after coming home	0.06	0.02	0.0008
Washing hands before eating	0.02	0.02	0.3655
Washing hands after toilet	0.04	0.02	0.0323
Washing hands after contact with animals	0.04	0.02	0.0171
Owing a cat	0.01	0.02	0.4801
Owing a dog	-0.03	0.02	0.077
Owing a cat and a dog	0	0.02	0.7061
*Helicobacter pylori* infection	-0.03	0.02	0.0602

**Table 3:** Multiple regression models for HAZ, WAZ, and BMIZ.

Variable	*R* ^2^	Effect	*β* stand	SE *β* stand	95% CI		*p*
HAZ	0.010	Washing hands after toilet	0.05	0.02	0.00	0.07	0.0203
WAZ	0.017	Male sex	0.09	0.02	0.05	0.12	<0.0001
		Living in the old town district	-0.05	0.02	-0.09	-0.02	0.0039
		Using collective catering facilities	-0.08	0.02	-0.11	-0.04	<0.0001
		*Helicobacter pylori* infection	-0.04	0.02	-0.08	-0.04	0.0269
BMIZ	0.025	Male sex	0.12	0.02	0.08	0.15	<0.0001
		Consumption of unboiled water	0.06	0.02	0.02	0.09	0.0009
		Using collective catering facilities	-0.09	0.02	-0.12	-0.05	<0.0001
		*Helicobacter pylori* infection	-0.04	0.02	0.07	0.00	0.0265

**Table 4:** Unadjusted parameter estimate (*β*) with standard errors (SE) of WAZ according to sociodemographic and hygienic characteristics.

Variable	*β*	SE	*p*
Male sex	0.09	0.02	<0.0001
Age			
13 years	0	0.02	0.7748
14 years	-0.07	0.18	0.7048
15 years	-0.15	0.18	0.4248
16 years	-0.06	0.05	0.2791
17 years	0	0.02	0.9000
Living in the old town district	-0.05	0.02	0.0066
Consumption of raw vegetables	0.02	0.02	0.1518
Consumption of raw meat	0	0.02	0.9486
Consumption of unboiled water	0.03	0.02	0.0787
Using collective catering facilities	-0.06	0.02	0.0003
Washing hands after coming home	0	0.02	0.9453
Washing hands before eating	-0.02	0.02	0.3587
Washing hands after toilet	-0.01	0.02	0.4759
Washing hands after contact with animals	0.02	0.02	0.235
Owing a cat	0.01	0.02	0.6879
Owing a dog	-0.01	0.02	0.4488
Owing cat and dog	0	0.02	0.2791
*Helicobacter pylori* infection	-0.05	0.02	0.0085

**Table 5:** Unadjusted parameter estimate (*β*) with standard errors (SE) of BMIZ according to sociodemographic and hygienic characteristics.

Variable	*β*	SE	*p*
Male sex	0.11	0.02	<0.0001
Age			
13 years	0.01	0.02	0.3621
14 years	-0.01	0.19	0.9253
15 years	-0.08	0.18	0.6642
16 years	-0.05	0.01	0.8616
17 years	0	0.02	0.8931
Living in the old town district	-0.02	0.02	0.2323
Consumption of raw vegetables	0.02	0.02	0.2746
Consumption of raw meat	0.01	0.02	0.4275
Consumption of unboiled water	0.05	0.02	0.0017
Using collective catering facilities	-0.07	0.02	0.0001
Washing hands after coming home	-0.03	0.02	0.0837
Washing hands before meal	-0.03	0.02	0.1148
Washing hands after toilet	-0.03	0.02	0.0573
Washing hands after contact with animals	0	0.02	0.9243
Owing a cat	0	0.02	0.9317
Owing a dog	0	0.02	0.9571
Owing a cat and a dog	0	0.01	0.7241
*Helicobacter pylori* infection	-0.04	0.02	0.0246

## Data Availability

The data to support the findings of this study are available on request from the corresponding author (ASzP).
